# The influence of different current-intensity transcranial alternating current stimulation on the eyes-open and eyes-closed resting-state electroencephalography

**DOI:** 10.3389/fnhum.2022.934382

**Published:** 2022-08-17

**Authors:** Yao Wang, Peiyun Hou, Wenjing Li, Mingxing Zhang, Hongliang Zheng, Xiaogang Chen

**Affiliations:** ^1^School of Life Sciences, Tiangong University, Tianjin, China; ^2^School of Precision Instruments and Optoelectronics Engineering, Tianjin University, Tianjin, China; ^3^Institute of Biomedical Engineering, Chinese Academy of Medical Sciences and Peking Union Medical College, Tianjin, China

**Keywords:** transcranial alternating current stimulation, electroencephalography, resting state, alpha oscillation, visual cortex

## Abstract

Transcranial alternating current stimulation (tACS) applies a sinusoidal oscillating current to modulate intrinsic oscillatory activity. Relevant studies of tACS have indicated that tACS can increase spontaneous brain activity in the occipital area. However, few studies have compared the effects of tACS with different current intensities on spontaneous brain activity in the occipital region. In this study, 10-Hz tACS was delivered to the occipital region at different current intensities (i.e., 1 and 2 mA). We investigated the effect of the tACS on both eyes-open and eyes-closed resting-state electroencephalography (EEG). A total of 20 subjects and fifteen subjects were recruited to participate in the 1-mA tACS experiment and the 2-mA tACS experiment, respectively. Ten subjects participated in both experiments. The experimental results demonstrated that both 1-mA tACS and 2-mA tACS could increase occipital resting-state EEG activities. For the eyes-open condition, alpha activity elicited by 2-mA tACS increased significantly greater than that elicited by 1-mA tACS, while 1-mA tACS could produce greater alpha activity compared to 2 mA for the eyes-closed condition. These results suggested that the optimal current intensity might be different for the eyes-open and eyes-closed resting-state conditions, laying a foundation for the subsequent study of occipital tACS on task-state EEG activities.

## Introduction

Due to the excellent plasticity of the visual cortex (Levelt and Hübener, 2012), numerous studies have been conducted to explore the brain oscillation activity in the visual cortex. However, the visual excitability was lower in some subjects due to individual variability. To improve the brain oscillatory excitability in the visual cortex, the investigators used transcranial alternating current stimulation (tACS) as a treatment approach to improve neural response in the visual cortex (Schuhmann et al., 2019; Ronconi et al., 2020). tACS is an important method that can be used to mimic the alternating nature of brain oscillations and regulate intrinsic oscillatory activity through sinusoidal oscillatory currents (Klink et al., 2020). Due to its non-invasive characteristics (Yavari et al., 2018), tACS has been widely applied to study various topics in neuroscience (Kasten et al., 2019), such as working memory (Nikolin et al., 2018; Abellaneda-Pérez et al., 2019) and visual attention (Hopfinger et al., 2017; de Graaf et al., 2020). Thus, tACS can be used as an important means of cognitive regulation.

Transcranial alternating current stimulation can regulate electrical signals from the visual cortex by changing the parameters such as frequency, current intensity, phase (Fiene et al., 2020), and stimulation duration (Vosskuhl et al., 2018). It can also modulate the rhythmic activity of the brain regions of interest in a frequency-dependent manner (Zaehle et al., 2010). For example, D’Atri et al. (2019) studied the effects of 5-Hz tACS on resting-state EEG signals and found that 5-Hz tACS increased frontotemporal signals during both wakefulness and sleepiness. Helfrich et al. (2014) used 10-Hz tACS to increase alpha activity in the parietal-occipital visual cortex. Other studies have also demonstrated that the modulation frequency of the visual area by tACS was mainly concentrated in the alpha band (Iemi et al., 2017; Klink et al., 2020). Therefore, we chose 10 Hz in the alpha band with the strongest response in the visual region as the frequency of tACS.

In previous studies, researchers also have used different current intensities to explore the effect of tACS on visual cortex responses. For example, Duan and Zhang studied the effect of tACS (1 mA) on improving brain–computer interface performance based on steady-state visual evoked potential (SSVEP) (Duan and Zhang, 2016). They found that 1-mA tACS had a positive effect on SSVEP and increased the brain electrical activity in the occipital region. Dowsett et al. (2020) used tACS with different current intensities (peak-to-peak value: 0.2, 1, and 2 mA) and frequencies (10, 8.5, and 12.5 Hz) to study the effects on illusory self-movement induced by large optical current stimulation. They analyzed the reliable influence of tACS on frequency-matching SSVEP. Their results showed that tACS with 2-mA intensity enhanced the visual evoked response of the occipital region more effectively than a lower stimulation intensity. Therefore, the previous studies have proved that both 1- and 2-mA tACS can enhance the occipital visual cortex signals in a task state.

Research on resting-state cortical signals can also provide valuable insights into the study of neuroscience and diseases (Newson and Thiagarajan, 2018; Clarke et al., 2020). Zarubin et al. (2020) studied the modulation of the brain electrical activity in the resting visual system using 1-mA short-term tACS. The results showed that the alpha oscillation after tACS increased in both the eyes-open and eyes-closed states, although the alpha oscillation was generally higher in the eyes-closed state. Similarly, Neuling et al. (2015) and Fuscà et al. (2018) used the magnetoencephalogram to study the modulation effects of tACS on the alpha oscillation of the visual cortex by functional network changes and amplitude changes, respectively. They both applied tACS with two stimulus intensities (strong tACS: as low as 0.1 mA and as high as 1.5 mA; weak tACS: 0.05 mA) to the occipital region in the eyes-open and eyes-closed states. Their results showed that the spontaneous brain activity in the occipital region increased in the two stimulus conditions. Furthermore, stronger tACS produces greater spontaneous brain activity in the eyes-open and eyes-closed resting states. These studies verified the enhancement effect of tACS on spontaneous brain activity in the resting occipital visual cortex. However, they did not study in detail whether the state-dependent effect of tACS will change with the current intensities. The state-dependent effect refers to the different modulation effects of tACS on EEG signals in two resting states of eyes open and eyes closed. Therefore, this study aimed to investigate the effects of 1- and 2-mA tACS on the resting EEG signals during eyes-open and eyes-closed states with the visual cortex as the target area. We used 1- and 2-mA tACS which have been shown to effectively modulate the spontaneous brain activity in the occipital region (Hosseinian et al., 2021). This study was used to explore the stimulation parameters that could further enhance the spontaneous brain activity of resting EEG signals in the visual cortex.

## Materials and methods

### Participants

A total of 25 healthy subjects who had normal vision or normal vision after correction took part in this study. Among them, 20 subjects (age: 23 ± 3 years, 6 males) participated in the low stimulus intensity (1 mA) experiment and 15 subjects (age: 23.5 ± 3.5 years, 7 males) participated in the high stimulus intensity (2 mA) experiment. Furthermore, 10 subjects participated in both low and high stimulus intensity experiments. All subjects provided written informed consent and received financial compensation for their participation. This study was approved by the Institution Review Board of Tsinghua University.

### Electroencephalography recording

In this study, EEG signals were collected using a 64-channel Neuroscan SynAmps 2 system. All electrodes were placed according to the international 10–20 system. The reference and ground electrodes were mounted on the vertex and between FPz and Fz, respectively. The sampling frequency of EEG was 1,000 Hz, and the band-pass filtering range was from 0.15 to 200 Hz. The system filtered out the 50 Hz power-line interference online. The impedance of all electrodes was kept below 10 kΩ.

### Transcranial alternating current stimulation

**FIGURE 1:**
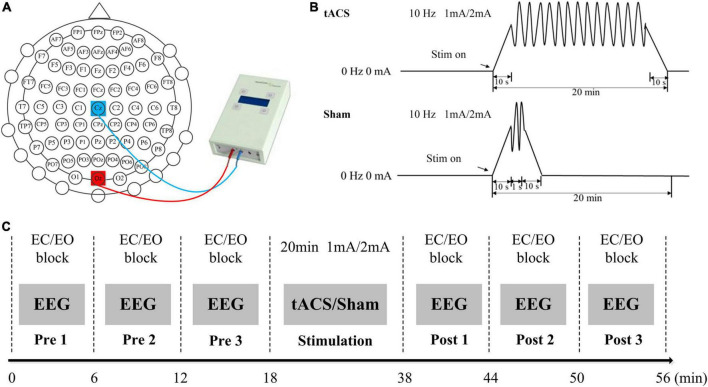
Experimental design. **(A)** The positions of the stimulating electrodes. **(B)** Experimental stimulation. **(C)** Experimental paradigm. EO, during the eyes-open state; EC, during the eyes-closed state.

Transcranial alternating current stimulation was applied using a NeuroConn DC-STIMULATOR. The subjects were electrically stimulated by a pair of saline-soaked sponge electrodes (5 cm × 7 cm). The two sponge electrodes were placed at the Cz and Oz electrodes (Figure 1A); the positions of the two electrodes could obtain the maximum stimulation intensity in the occipital region (Neuling et al., 2012). There were three stimulation conditions with a frequency of 10 Hz (Figure 1B): (1) sham stimulation that the current lasted only for 1 s after being raised to 1 or 2 mA; (2) tACS with 1-mA current intensity; and (3) tACS with 2-mA current intensity. For each condition, stimulation lasted for 20 min, with 10-s ramping up and down at the beginning and the end of the stimulation period.

### Experimental design

Two experiments were performed, namely, (1) low stimulation intensity experiment and (2) high stimulation intensity experiment. The interval between the two experiments was at least 1 month. Both the low stimulation intensity experiment and the high stimulation intensity experiment involved two experimental groups, namely, tACS and sham stimulation (1 or 2 mA). For both experiments, the two conditions (tACS and sham) were separated by at least 1 week.

Subjects sat in a comfortable position and were told to keep quiet during the experiments. The entire experiments were carried out in an electromagnetically shielded room. Each experiment included a pre-stimulation EEG recording part, a stimulation part, and a post-stimulation EEG recording part (Figure 1C). There were three blocks both in pre- and post-stimulation EEG recording parts. One block included 18 eyes-open and 18 eyes-closed tasks. The subjects changed the eyes-open and eyes-closed states according to the voice prompts and each resting-state EEG data lasted for 10 s. In this study, the stimulus program was developed under MATLAB using the Psychophysics Toolbox Version 3. A white fixation cross (80 × 80 pixels) was presented at the center of the screen during the eyes-open resting recording. In this study, subjects were asked to focus attention on the white fixation cross during the eyes-open resting recording, which was similar to the experimental design of Zaehle et al.’s (2010) study. This task might help keep subjects in a similar state for easy comparison. In addition, the subjects kept awake during the eyes-closed state. The total time for each block was 6 min. Between the adjacent blocks, subjects took a 1–2 min break. During the stimulation part, the subjects were exposed to tACS or sham stimulation with an intensity of 1 or 2 mA for 20 min. The sequence of tACS and sham stimulation was randomly determined. All subjects were blinded to the stimulation design.

### Data analysis

The collected EEG signals were referenced averagely to reduce the influence of noise and improve the signal-to-noise ratio (SNR). Subsequently, the re-referenced data were filtered with a band-pass filter of 0.5–49 Hz to remove unwanted low- and high-frequency signal interference. The data were divided into 10-s data segments for both the eyes-open and eyes-closed states. The 200-ms data before the stimulation were used as the baseline for baseline correction. In addition, further independent component analysis (ICA) was used to remove EEG artifacts. Subsequently, the data were divided into 4-s epochs that overlapped by 50% for both eyes-open and eyes-closed states (Dong et al., 2020). Fast Fourier transform (FFT) spectra were computed and averaged for all epochs.

To determine the frequency bands where brain activity was most active, the number of electrodes which had a significant effect on the amplitude between pre- and post-stimulation was calculated under both tACS and sham stimulation in each frequency band. The modulation area of tACS was determined by comparing the amplitude maps and the topographic maps. The paired *t*-test was conducted between pre- and post-stimulation in both eyes-open and eyes-closed states to investigate the effect of different current intensities of tACS and sham stimulation. We performed a repeated-measures ANOVA including two factors, i.e., stimulation types (tACS and sham stimulation) and stimulation times (pre-stimulation and post-stimulation) in order to demonstrate a difference between tACS and sham stimulation. In this study, a total of 10 subjects participated in the experiment under two current strengths. In order to analyze the effect of different current intensities of tACS on endogenous alpha oscillations during the eyes-open and eyes-closed states, we used repeated-measures ANOVA including two factors, i.e., resting states (eyes open and eyes closed) and current intensities (1-mA tACS and 2-mA tACS).

## Results

**FIGURE 2:**
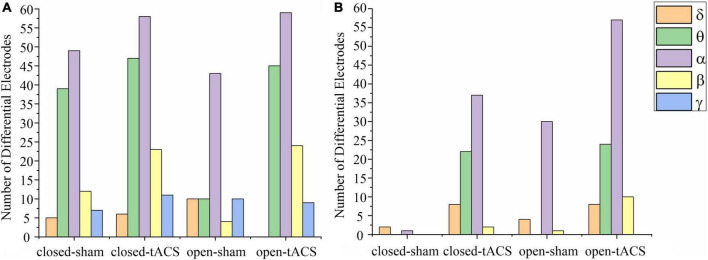
The number of electrodes has a significant difference between pre-and post-stimulation within different frequency bands under each experimental condition. **(A)** The stimulation intensity was 1 mA. **(B)** The stimulation intensity was 2 mA. The number of significantly different electrodes at the blank position is zero. Closed-sham, under the sham stimulation in the eyes-closed state; closed-tACS, under the tACS in the eyes-closed state; open-sham, under the sham stimulation in the eyes-open state; open-tACS, under the tACS in the eyes-open state.

**FIGURE 3:**
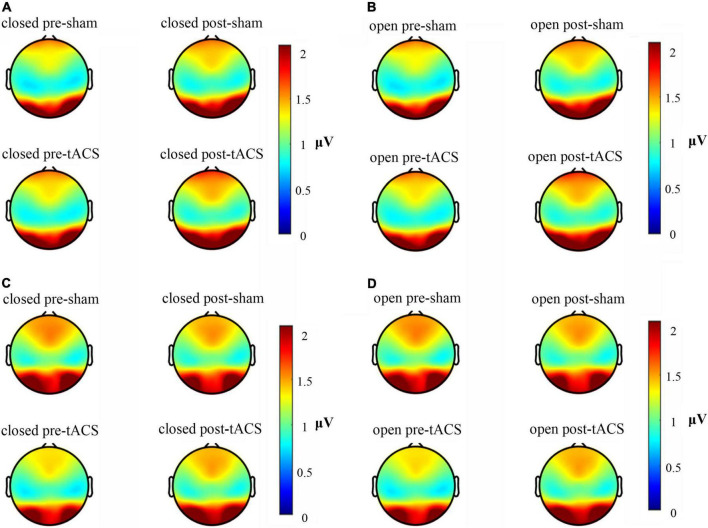
Brain electrical activity mappings in the alpha frequency band under different stimulation conditions. **(A)** Topographic map of 1-mA current intensity with the eyes-closed condition. **(B)** Topographic map of 1-mA current intensity with the eyes-open condition. **(C)** Topographic map of 2-mA current intensity with the eyes-closed condition. **(D)** Topographic map of 2-mA current intensity with the eyes-open condition.

The available frequency spectrum was first divided as follows: delta (0.5–4 Hz), theta (4–8 Hz), alpha (8–12 Hz), beta (12–30 Hz), and gamma (30–49 Hz). In each experimental condition, there were a maximum number of electrodes in the alpha band (Figure 2). It indicated that the brain cortical response was strongest in the alpha band. Therefore, follow-up analysis was focused on alpha-band data. As shown in Figure 3, the main response of the visual cortex in the alpha frequency band was in the occipital region, and the main changes after stimulation were within the six electrodes of the occipital region, namely, O1, Oz, O2, PO3, POz, and PO4. Moreover, the alpha endogenous activity in the occipital region after 1- and 2-mA tACS was enhanced in topographic maps.

**FIGURE 4:**
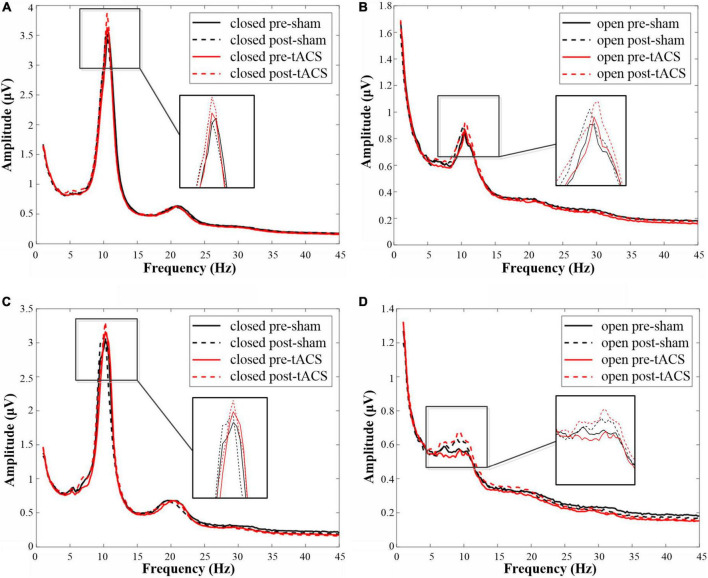
The amplitudes in the alpha frequency band under each condition. **(A)** The amplitude map of 1-mA stimulation in the eyes-closed state. **(B)** The amplitude map of 1-mA stimulation in the eyes-open state. **(C)** The amplitude map of 2-mA stimulation in the eyes-closed state. **(D)** The amplitude map of 2-mA stimulation in the eyes-open state.

**FIGURE 5:**
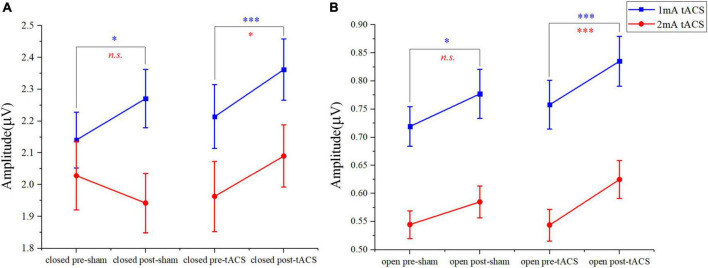
The paired *t*-test results in amplitudes between post- and pre-stimulation for different intensities of tACS and sham stimulation. **(A)** The results of pre-sham, post-sham, pre-tACS, and post-tACS with the eyes-closed condition. **(B)** The results of pre-sham, post-sham, pre-tACS, and post-tACS with the eyes-open condition; *n.s.* > 0.05, *p < 0.05, ^***^p < 0.001.

In the eyes-closed state, the amplitude in the alpha band was increased both after tACS and sham stimulation with an intensity of 1 mA. Among them, the amplitude after 1-mA tACS was increased the most (Figure 4A). A paired *t*-test indicated that the amplitude was different significantly between post- and pre-stimulation for both 1-mA tACS and sham conditions (Figure 5A, tACS: *p* < 0.001; sham: *p* = 0.04). To verify the effect of tACS compared to the sham stimulation, we compared the amplitude between the tACS and sham conditions. The repeated-measures ANOVA results revealed that the alpha amplitude between the tACS group and the sham group had a significant difference with 1-mA stimulation [*F*(1,38) = 9.136, *p* = 0.003]. For the intensity of 2 mA, the paired *t*-test results revealed that sham stimulation had no significant effect on the amplitude in the alpha band (Figures 4C, 5A, *p* = 0.131). However, tACS increased alpha band amplitude significantly post-tACS compared to the pre-tACS (Figures 4C, 5A, *p* = 0.012). The repeated-measures ANOVA results revealed that the alpha amplitude between the tACS group and the sham group had a significantly different effect on the alpha amplitude with 2-mA stimulation [*F*(1,28) = 3.248, *p* < 0.001].

In the eyes-open state, amplitude in the alpha band after 1-mA stimulations shows a similar trend as in the eyes-closed state. Both tACS and sham stimulation increased the amplitude slightly, and the amplitude after tACS increased more (Figure 4B). The paired *t*-test results indicated that there was a significant difference in amplitudes between post- and pre-stimulation for both tACS and sham stimulation (Figure 5B, sham: *p* = 0.033; tACS: *p* < 0.001). The repeated-measures ANOVA results revealed that the alpha amplitude between the tACS group and the sham group had a significantly different effect on the alpha amplitude with 1-mA stimulation [*F*(1,38) = 12.570, *p* < 0.001]. For the intensity of 2 mA, although the alpha amplitude increased slightly after sham stimulation (Figure 4D), the paired *t*-test results revealed that sham stimulation had no significant effect (Figure 5B, *p* = 0.056). However, tACS increased alpha band amplitude significantly post-tACS compared to the pre-tACS (Figure 5B, *p* < 0.001). The repeated-measures ANOVA results revealed that the alpha amplitude between the tACS group and the sham group had a significantly different effect on the alpha amplitude with 2-mA stimulation [*F*(1,28) = 4.324, *p* = 0.039].

**FIGURE 6:**
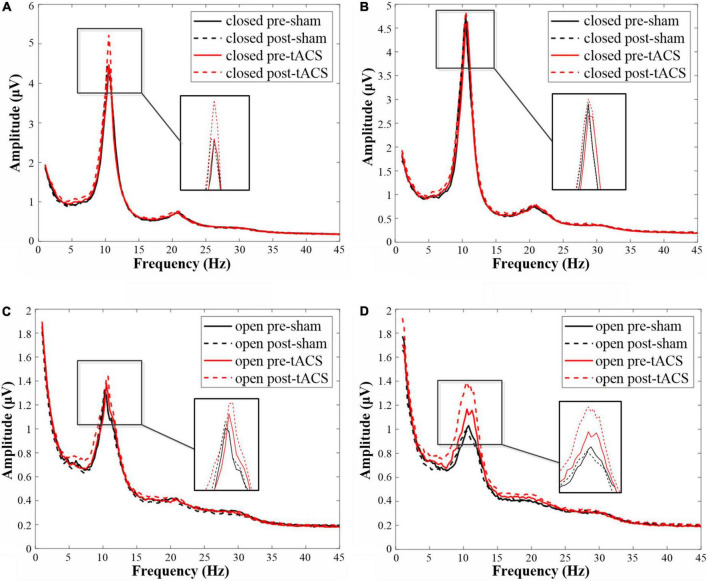
The amplitude in the alpha frequency band under each condition within 10 subjects who participated in both low- and high-stimulus experiments. **(A)** The amplitude map of 1-mA stimulation in the eyes-closed state. **(B)** The amplitude map of 1-mA stimulation in the eyes-open state. **(C)** The amplitude map of 2-mA stimulation in the eyes-closed state. **(D)** The amplitude map of 2-mA stimulation in the eyes-open state.

**FIGURE 7:**
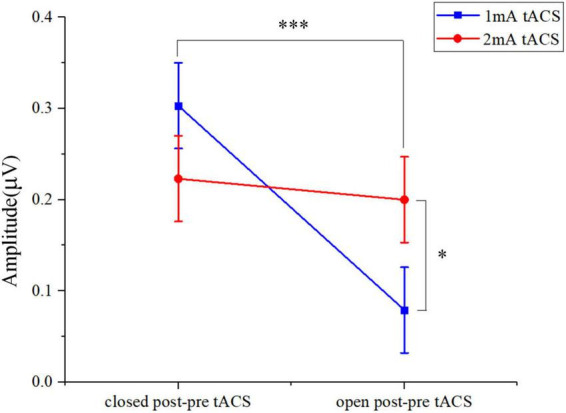
The two-way ANOVA results in alpha band amplitude under tACS condition within 10 subjects who participated in both 1- and 2-mA stimulation. Closed post-pre tACS, the alpha amplitude differences between the pre- and post-tACS in the eyes-closed state; open post-pre tACS, the alpha amplitude differences between the pre- and post-tACS in the eyes-open state; *p < 0.05, ^***^p < 0.001.

Both 1- and 2-mA tACS increased the alpha band amplitude within 10 subjects who participated in the two current intensities’ experiments under eyes-open and eyes-closed conditions (Figure 6). For the tACS part, the alpha amplitude after 1-mA tACS (0.303 ± 0.361, mean ± standard deviation) increased more than 2-mA tACS (0.223 ± 0.551; Figure 7) in the eyes-closed state, while in the eyes-open state, the alpha amplitude after 2-mA tACS (0.200 ± 0.292) increased more than 1-mA tACS (0.079 ± 0.131; Figure 7). The repeated-measures ANOVA results revealed that both current intensities and resting states had a significant effect on the alpha amplitude differences between the pre- and post-tACS [current intensities: *F*(1,18) = 4.159, *p* = 0.044; resting states: *F*(1,18) = 7.463, *p* = 0.007]. In addition, there was no statistically significant interaction between current intensities and resting states [*F*(1,18) = 0.204, *p* = 0.652].

## Discussion

This study aimed to elucidate the effect of different intensities of tACS on spontaneous brain activity in the visual cortex. The results revealed that both 1- and 2-mA tACS enhanced the occipital visual cortex response in the eyes-open and eyes-closed states. The response was strongest in the alpha frequency band. Moreover, these results also found that the 1-mA tACS enhanced the alpha endogenous activity in the occipital region more than the 2-mA tACS in the eyes-closed state. However, in the eyes-open state, the enhanced occipital response trend at 2 mA was more significant than that at 1 mA.

This study demonstrated that both 1- and 2-mA tACS enhanced spontaneous brain activity in the occipital visual cortex, especially in the alpha band amplitude. In contrast to previous studies, our results are consistent with the past results that tACS enhances the resting-state alpha oscillation (Zaehle et al., 2010; Ruhnau et al., 2016). Nakazono et al. (2020) studied that alpha tACS was effective for visual functions. The tACS in the alpha band was more able to enhance alpha oscillations in spontaneous brain activity (Ghiani et al., 2021). Therefore, the main area of the alpha tACS effect was the visual cortex area in the resting states. In previous studies, 1.5 mA was the highest current intensity of tACS shown to enhance the resting-state signal in the visual cortex (Neuling et al., 2013). However, this study demonstrated that the highest current intensity of tACS which enhanced the effect on the resting-state response of the visual cortex can reach 2 mA.

In this study, the resting-state response in the visual cortex with the eyes-closed condition was more sensitive to a current intensity of 1 mA. This result is consistent with previous studies (Neuling et al., 2015; Zarubin et al., 2020). However, Neuling et al. (2013) showed that alpha amplitude did not differ significantly between post- and pre-stimulation for tACS with an initial intensity of 1.5 mA. Nunez et al. (2001) reported that the endogenous alpha spontaneous brain activity was not further enhanced after reaching the maximum value, indicating a ceiling effect. Therefore, the alpha amplitude of spontaneous brain activity in the eyes-closed state may have been enhanced to its maximum value, i.e., 1-mA tACS. However, higher intensity tACS no longer enhanced the alpha endogenous oscillation of the occipital region.

We further found that 2-mA tACS enhanced the alpha endogenous oscillation of the resting-state occipital area in the eyes-open condition. Most previous studies have indicated that 1- or 1.5-mA tACS increased the alpha endogenous oscillation in the eyes-open state (Zaehle et al., 2010; Kasten et al., 2016). The eyes-open state, which has low endogenous oscillation, was more sensitive to changes in tACS current intensity (Barry et al., 2007). Therefore, we speculate that the enhancement of the tACS current intensity within the range from 1 to 2 mA may lead to the enhancement of the alpha endogenous oscillation in the eyes-open state. Thus, when studying the effect of tACS on task-state EEG activities, researchers could enhance the highest current intensity of tACS with 2 mA to improve SSVEP.

Overall, there were differences in the enhancement effect of 1- and 2-mA tACS on the resting-state occipital region alpha response between the eyes-open and eyes-closed states. Barry et al. (2007) found that the eyes-closed and eyes-open conditions provided different topographical maps and brain activity measurements at different power levels in the resting state. The influence of tACS on the amplitude of EEG would change at different brain states (Fuscà et al., 2018). Ruhnau et al.’s (2016) study demonstrated that tACS with individual alpha frequency entrained brain activity in visual cortices. This effect is state-dependent and is clearly observed with eyes open but only to a lesser extent with eyes closed. The eye-closed resting state has high endogenous oscillations, and the eye-open resting state has low endogenous oscillations. Compared with high endogenous oscillations, low endogenous oscillations are more likely to enhance alpha oscillations in the occipital region under high current-intensity tACS. Therefore, the effect of tACS with different current intensities may differ under the different resting states.

One limitation of our study is that we only examined the effects of tACS on the resting visual cortex. Subsequent research could apply the optimal parameters of tACS identified here to study the effect of tACS on the brain–computer interface (BCI) of SSVEP. Another limitation concerns that tACS was applied to identical current intensity for all subjects at each stimulation intensity condition. Due to individual differences (Kasten et al., 2019), the standard stimulation paradigm does not fit all subjects well. The effect of 2-mA tACS on resting visual cortical responses may vary across individual subjects. Therefore, future studies could find the optimal current strength for the individual subject in the 1–2 mA range.

## Conclusion

In this study, we investigated the effect of the tACS on both eyes-open and eyes-closed resting-state EEG. Results obtained from 25 healthy subjects revealed that the resting-state response in the visual cortex with the eyes-closed condition was more sensitive to a current intensity of 1 mA. Furthermore, for the eyes-open state, alpha activity elicited by 2-mA tACS increased significantly greater than that elicited by 1-mA tACS. These results suggest that the effect of different current intensities differs between the eyes-open and eyes-closed states. This study presents evidence for the subsequent study of occipital tACS on task-state EEG activities.

## Data availability statement

The raw data supporting the conclusions of this article will be made available by the authors, without undue reservation.

## Ethics statement

The studies involving human participants were reviewed and approved by the Institution Review Board of Tsinghua University. The patients/participants provided their written informed consent to participate in this study.

## Author contributions

YW and XC: experimental design and manuscript revision. PH: experimental design and operation, data analysis, and manuscript writing. WL: experimental operation and data analysis. HZ and MZ: experimental operation and data collection. All authors contributed to the article and approved the submitted version.

## Conflict of interest

The authors declare that the research was conducted in the absence of any commercial or financial relationships that could be construed as a potential conflict of interest.

## Publisher’s note

All claims expressed in this article are solely those of the authors and do not necessarily represent those of their affiliated organizations, or those of the publisher, the editors and the reviewers. Any product that may be evaluated in this article, or claim that may be made by its manufacturer, is not guaranteed or endorsed by the publisher.

## References

[B1] Abellaneda-Pérez K. Vaqué-Alcázar L. Perellón-Alfonso R. Bargalló N. Kuo M. F. Pascual-Leone A. (2019). Differential tDCS and tACS effects on working memory-related neural activity and resting-state connectivity. *Front. Neurosci.* 13:1440. 10.3389/fnins.2019.01440 32009896 PMC6978675

[B2] Barry R. J. Clarke A. R. Johnstone S. J. Magee C. A. Rushby J. A. (2007). EEG differences between eyes-closed and eyes-open resting conditions. *Clin. Neurophysiol.* 118 2765–2773. 10.1016/j.clinph.2007.07.028 17911042

[B3] Clarke A. R. Barry R. J. Johnstone S. (2020). Resting state EEG power research in Attention-Deficit/Hyperactivity disorder: a review update. *Clin. Neurophysiol.* 131 1463–1479. 10.1016/j.clinph.2020.03.029 32387965

[B4] D’Atri A. Scarpelli S. Gorgoni M. Alfonsi V. Annarumma L. Giannini A. M. (2019). Bilateral theta Transcranial Alternating Current Stimulation (tACS) modulates EEG activity: when tACS Works awake it also works asleep. *Nat. Sci. Sleep* 11 343–356. 10.2147/nss.S229925 31819688 PMC6875492

[B5] de Graaf T. A. Thomson A. Janssens S. E. W. van Bree S. Ten Oever S. Sack A. T. (2020). Does alpha phase modulate visual target detection? Three experiments with tACS-phase-based stimulus presentation. *Eur. J. Neurosci.* 51 2299–2313. 10.1111/ejn.14677 31943418 PMC7317496

[B6] Dong G. Wang Y. Chen X. (2020). Anodal occipital tDCS enhances spontaneous alpha activity. *Neurosci. Lett.* 721:134796. 10.1016/j.neulet.2020.134796 32006627

[B7] Dowsett J. Herrmann C. S. Dieterich M. Taylor P. C. J. (2020). Shift in lateralization during illusory self-motion: EEG responses to visual flicker at 10 Hz and frequency-specific modulation by tACS. *Eur. J. Neurosci.* 51 1657–1675. 10.1111/ejn.14543 31408562

[B8] Duan R. Zhang D. (2016). “Effects of transcranial alternating current stimulation on performance of SSVEP-based brain-computer interface,” in *IEEE International Conference on Real-time Computing & Robotics*, (Angkor Wat).

[B9] Fiene M. Schwab B. C. Misselhorn J. Herrmann C. S. Schneider T. R. Engel A. K. (2020). Phase-specific manipulation of rhythmic brain activity by transcranial alternating current stimulation. *Brain Stimul.* 13, 1254–1262. 10.1016/j.brs.2020.06.008 32534253

[B10] Fuscà M. Ruhnau P. Neuling T. Weisz N. (2018). Local network-level integration mediates effects of transcranial alternating current stimulation. *Brain Connect.* 8 212–219. 10.1089/brain.2017.0564 29478338

[B11] Ghiani A. Maniglia M. Battaglini L. Melcher D. Ronconi L. (2021). Binding mechanisms in visual perception and their link with neural oscillations: a review of evidence from tACS. *Front. Psychol.* 12:643677. 10.3389/fpsyg.2021.643677 33828509 PMC8019716

[B12] Helfrich R. F. Schneider T. R. Rach S. Trautmann-Lengsfeld S. A. Engel A. K. Herrmann C. S. (2014). Entrainment of brain oscillations by transcranial alternating current stimulation. *Curr. Biol.* 24 333–339. 10.1016/j.cub.2013.12.041 24461998

[B13] Hopfinger J. B. Parsons J. Fröhlich F. (2017). Differential effects of 10-Hz and 40-Hz transcranial alternating current stimulation (tACS) on endogenous versus exogenous attention. *Cogn. Neurosci.* 8 102–111. 10.1080/17588928.2016.1194261 27297977

[B14] Hosseinian T. Yavari F. Biagi M. C. Kuo M. F. Ruffini G. Nitsche M. A. (2021). External induction and stabilization of brain oscillations in the human. *Brain Stimul.* 14 579–587. 10.1016/j.brs.2021.03.011 33781955 PMC8144019

[B15] Iemi L. Chaumon M. Crouzet S. M. Busch N. A. (2017). Spontaneous neural oscillations bias perception by modulating baseline excitability. *J. Neurosci.* 37 807–819. 10.1523/jneurosci.1432-16.2017 28123017 PMC6597018

[B16] Kasten F. H. Dowsett J. Herrmann C. S. (2016). Sustained aftereffect of α-tACS lasts up to 70 min after stimulation. *Front. Hum. Neurosci.* 10:245. 10.3389/fnhum.2016.00245 27252642 PMC4879138

[B17] Kasten F. H. Duecker K. Maack M. C. Meiser A. Herrmann C. S. (2019). Integrating electric field modeling and neuroimaging to explain inter-individual variability of tACS effects. *Nat. Commun.* 10:5427. 10.1038/s41467-019-13417-6 31780668 PMC6882891

[B18] Klink K. Paßmann S. Kasten F. H. Peter J. (2020). The modulation of cognitive performance with transcranial alternating current stimulation: a systematic review of frequency-specific effects. *Brain Sci.* 10:932. 10.3390/brainsci10120932 33276533 PMC7761592

[B19] Levelt C. N. Hübener M. (2012). Critical-period plasticity in the visual cortex. *Annu. Rev. Neurosci.* 35 309–330. 10.1146/annurev-neuro-061010-113813 22462544

[B20] Nakazono H. Ogata K. Takeda A. Yamada E. Kimura T. Tobimatsu S. (2020). Transcranial alternating current stimulation of α but not β frequency sharpens multiple visual functions. *Brain Stimul.* 13 343–352. 10.1016/j.brs.2019.10.022 31711878

[B21] Neuling T. Rach S. Herrmann C. S. (2013). Orchestrating neuronal networks: sustained after-effects of transcranial alternating current stimulation depend upon brain states. *Front. Hum. Neurosci.* 7:161. 10.3389/fnhum.2013.00161 23641206 PMC3639376

[B22] Neuling T. Ruhnau P. Fuscà M. Demarchi G. Herrmann C. S. Weisz N. (2015). Friends, not foes: magnetoencephalography as a tool to uncover brain dynamics during transcranial alternating current stimulation. *Neuroimage* 118 406–413. 10.1016/j.neuroimage.2015.06.026 26080310 PMC4686537

[B23] Neuling T. Wagner S. Wolters C. H. Zaehle T. Herrmann C. S. (2012). Finite-element model predicts current density distribution for clinical applications of tDCS and tACS. *Front. Psychiatry* 3:83. 10.3389/fpsyt.2012.00083 23015792 PMC3449241

[B24] Newson J. J. Thiagarajan T. C. (2018). EEG frequency bands in psychiatric disorders: a review of resting state studies. *Front. Hum. Neurosci.* 12:521. 10.3389/fnhum.2018.00521 30687041 PMC6333694

[B25] Nikolin S. Martin D. Loo C. K. Boonstra T. W. (2018). Effects of TDCS dosage on working memory in healthy participants. *Brain Stimul.* 11 518–527. 10.1016/j.brs.2018.01.003 29361442

[B26] Nunez P. L. Wingeier B. M. Silberstein R. B. (2001). Spatial-temporal structures of human alpha rhythms: theory, microcurrent sources, multiscale measurements, and global binding of local networks. *Hum. Brain Mapp.* 13 125–164. 10.1002/hbm.1030 11376500 PMC6872048

[B27] Ronconi L. Melcher D. Junghofer M. Wolters C. H. Busch N. A. (2020). Testing the effect of tACS over parietal cortex in modulating endogenous alpha rhythm and temporal integration windows in visual perception. *Eur. J. Neurosci*. 55, 3438–3450. 10.1111/ejn.15017 33098112 PMC9542321

[B28] Ruhnau P. Neuling T. Fuscá M. Herrmann C. S. Demarchi G. Weisz N. (2016). Eyes wide shut: transcranial alternating current stimulation drives alpha rhythm in a state dependent manner. *Sci. Rep.* 6:27138. 10.1038/srep27138 27252047 PMC4890046

[B29] Schuhmann T. Kemmerer S. K. Duecker F. de Graaf T. A. Ten Oever S. De Weerd P. (2019). Left parietal tACS at alpha frequency induces a shift of visuospatial attention. *PLoS One* 14:e0217729. 10.1371/journal.pone.0217729 31774818 PMC6881009

[B30] Vosskuhl J. Strüber D. Herrmann C. S. (2018). Non-invasive brain stimulation: a paradigm shift in understanding brain oscillations. *Front. Hum. Neurosci.* 12:211. 10.3389/fnhum.2018.00211 29887799 PMC5980979

[B31] Yavari F. Jamil A. Mosayebi Samani M. Vidor L. P. Nitsche M. A. (2018). Basic and functional effects of transcranial Electrical Stimulation (tES)-An introduction. *Neurosci. Biobehav. Rev.* 85 81–92. 10.1016/j.neubiorev.2017.06.015 28688701

[B32] Zaehle T. Rach S. Herrmann C. S. (2010). Transcranial alternating current stimulation enhances individual alpha activity in human EEG. *PLoS One* 5:e13766. 10.1371/journal.pone.0013766 21072168 PMC2967471

[B33] Zarubin G. Gundlach C. Nikulin V. Villringer A. Bogdan M. (2020). Transient amplitude modulation of alpha-band oscillations by short-time intermittent closed-loop tACS. *Front. Hum. Neurosci.* 14:366. 10.3389/fnhum.2020.00366 33100993 PMC7500443

